# 1-Formyl-*r*-2,*c*-6-bis­(4-methoxy­phen­yl)-*c*-3,*t*-3-dimethyl­piperidin-4-one

**DOI:** 10.1107/S1600536809010010

**Published:** 2009-03-25

**Authors:** T. Kavitha, S. Ponnuswamy, P. Sakthivel, K. Karthik, M. N. Ponnuswamy

**Affiliations:** a Centre of Advanced Study in Crystallography and Biophysics, University of Madras, Guindy Campus, Chennai 600 025, India; b Department of Chemistry, Government Arts College (Autonomous), Coimbatore 641 018, Tamilnadu, India

## Abstract

In the title compound, C_22_H_25_NO_4_, the piperidine ring adopts a distorted boat conformation. The two benzene rings are approximately perpendicular to each other, making a dihedral angle of 86.2 (8)°. The crystal packing is stabilized by C—H⋯O and C—H⋯π inter­actions.

## Related literature

For details of hydrogen-bond motifs, see: Bernstein *et al.* (1995 ▶). For ring conformational analysis, see: Cremer & Pople (1975 ▶); Nardelli (1983 ▶).
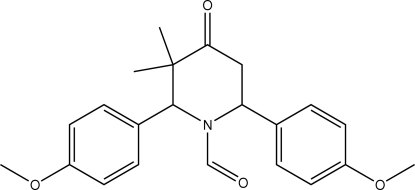


## Experimental

### 

#### Crystal data

C_22_H_25_NO_4_
*M* *_r_* = 367.43
Monoclinic, 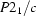

*a* = 11.7274 (3) Å
*b* = 18.8556 (4) Å
*c* = 9.7178 (3) Å
β = 113.507 (1)°
*V* = 1970.54 (9) Å^3^
*Z* = 4
Mo *K*α radiation
μ = 0.09 mm^−1^
*T* = 293 K
0.25 × 0.20 × 0.20 mm

#### Data collection

Bruker Kappa APEXII diffractometer
Absorption correction: multi-scan (*SADABS*; Sheldrick, 2001 ▶) *T*_min_ = 0.979, *T*_max_ = 0.983
26804 measured reflections
6250 independent reflections
4080 reflections with *I* > 2σ(*I*)
*R*_int_ = 0.031

#### Refinement

*R*[*F* ^2^ > 2σ(*F* ^2^)] = 0.049
*wR*(*F* ^2^) = 0.142
*S* = 1.03
6250 reflections
244 parameters
H-atom parameters constrained
Δρ_max_ = 0.23 e Å^−3^
Δρ_min_ = −0.19 e Å^−3^

### 

Data collection: *APEX2* (Bruker, 2004 ▶); cell refinement: *SAINT* (Bruker, 2004 ▶); data reduction: *SAINT*; program(s) used to solve structure: *SHELXS97* (Sheldrick, 2008 ▶); program(s) used to refine structure: *SHELXL97* (Sheldrick, 2008 ▶); molecular graphics: *PLATON* (Spek, 2009 ▶); software used to prepare material for publication: *SHELXL97* and *PARST* (Nardelli, 1983 ▶).

## Supplementary Material

Crystal structure: contains datablocks I, global. DOI: 10.1107/S1600536809010010/bt2903sup1.cif

Structure factors: contains datablocks I. DOI: 10.1107/S1600536809010010/bt2903Isup2.hkl

Additional supplementary materials:  crystallographic information; 3D view; checkCIF report

## Figures and Tables

**Table 1:** Hydrogen-bond geometry (Å, °)

*D*—H⋯*A*	*D*—H	H⋯*A*	*D*⋯*A*	*D*—H⋯*A*
C5—H5*A*⋯O3^i^	0.97	2.44	3.3446 (16)	155
C6—H6⋯O2^ii^	0.98	2.41	3.3708 (16)	168
C18—H18⋯O1^iii^	0.93	2.53	3.3018 (17)	140
C10—H10⋯*Cg*1^iv^	0.93	2.90	3.6627 (17)	140

Symmetry codes: (i) 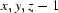; (ii) 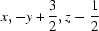; (iii) 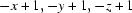; (iv) 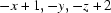. *Cg*(1) is the centroid of the C16–C21 ring.

**Fig. 1.**
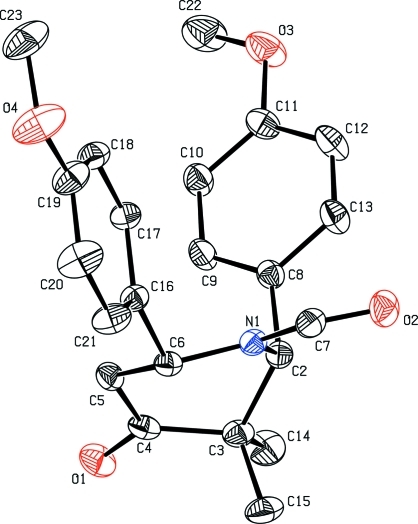
ORTEP plot of the molecule showing that the thermal ellipsoids are drawn at 30% probability level. H atoms have been omitted for clarity.

**Fig. 2.**
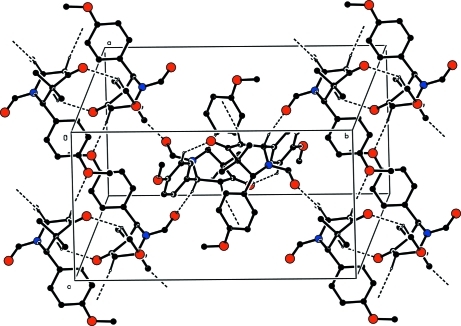
The molecular packing of the compound viewed down the b axis is shown. Dashed lines indicate hydrogen bonds. H atoms not involved in hydrogen bonding have been omitted.

## supplementary crystallographic information

### Comment

Piperidine, a basic component of the piper alkaloid piper nigrum is a monocyclic cyclohexane with a hetero atom affixed in the first position. The skeletal ring of piperidine is contained in the molecules of many synthetic and natural medicaments. A significant industrial application of piperidine is for the production of dipiperidinyl dithiuram tetrasulfide, which can be used as a rubber vulcanization accelerator.

The piperidine ring adopts distorted boat conformation with puckering parameters (Cremer & Pople, 1975) q2 = 0.630 (1) Å, q3 =0.070 (1)Å and φ2= 87.2 (1)° and the asymmetry parameters ΔC_2_(N1) and ΔC_2_(C4) = 14.78 (12)Å (Nardelli, 1983). The angles between the best plane of the piperidine ring (N1,C3,C4,C6) and the phenyl rings (C8—C13 and C16—C21) are 84.17 (7)° and 80.70 (7)°, respectively. The two phenyl rings are approximately perpendicular to each other as can be seen from the dihedral angle of 86.23 (8)°. The methyl substituents C14 and C15 are oriented equatorially [N1—C2—C3—C14 =] -178.88 (11)° and axially [N1—C2—C3—C15 =] -59.52 (13) ° with respect to the piperidine ring. The sum of the bond angles around N1 atom (359.3°) indicates *sp*^2^ hybridization.

The packing of the molecules is controlled by C—H···O types of intermolecular interactions. The symmetry related molecules form a dimer with the graph-set motif of *R*^2^_2_(16) (Bernstein *et al.*, 1995) through hydrogen bonds. Further a C—H··· π interaction also leads to the formation of a dimer [C10—H10 = 0.9301 Å, H10···*Cg*(1) =2.9035 Å, C10···*Cg*(1) = 3.6627 (17) Å and C10—H10···*Cg*(1) = 139.71°, where *Cg*(1) is the centroid of the ring (C16—C21) at (1 - *x*,-*y*,2 - *z*)] .

### Experimental

The ice-cold solution of acetic-formic anhydride was prepared from acetic anhydride (10 ml) and 85% formic acid (5 ml) and was added slowly to a cold solution of *r*-2, *c*-6-*bis*(4-methoxyphenyl)-*c*-3,*t*-3-dimethylpiperidine-4-one (1.69 g) in benzene (30 ml). The reaction mixture was stirred at room temperature for 5 hrs. The organic layer was separated, dried over anhydrous Na_2_SO_4_ and concentrated. The resulting mass was purified by crystallization from benzene-petroleum ether (333–353 K) in the ratio 1:1.

### Refinement

H atoms were positioned geometrically (C—H=0.93–0.96 Å) and allowed to ride on their parent atoms, with *U*_iso_(H) = 1.5*U*_eq_(C) for methyl H, 1.2*U*_eq_(C) for other H atoms.

### Crystal data

**Table:**

C_22_H_25_NO_4_	*F*(000) = 784
*M**_r_* = 367.43	*D*_x_ = 1.239 Mg m^−^^3^
Monoclinic, *P*2_1_/*c*	Mo *K*α radiation, λ = 0.71073 Å
Hall symbol: -P 2ybc	Cell parameters from 6250 reflections
*a* = 11.7274 (3) Å	θ = 2.2–31.0°
*b* = 18.8556 (4) Å	µ = 0.09 mm^−^^1^
*c* = 9.7178 (3) Å	*T* = 293 K
β = 113.507 (1)°	Block, colourless
*V* = 1970.54 (9) Å^3^	0.25 × 0.20 × 0.20 mm
*Z* = 4	

### Data collection

**Table:**

Bruker Kappa APEXII diffractometer	6250 independent reflections
Radiation source: fine-focus sealed tube	4080 reflections with *I* > 2σ(*I*)
graphite	*R*_int_ = 0.031
ω and φ scans	θ_max_ = 31.0°, θ_min_ = 2.2°
Absorption correction: multi-scan (*SADABS*; Sheldrick, 2001)	*h* = −16→16
*T*_min_ = 0.979, *T*_max_ = 0.983	*k* = −27→26
26804 measured reflections	*l* = −14→14

### Refinement

**Table:**

Refinement on *F*^2^	Primary atom site location: structure-invariant direct methods
Least-squares matrix: full	Secondary atom site location: difference Fourier map
*R*[*F*^2^ > 2σ(*F*^2^)] = 0.049	Hydrogen site location: inferred from neighbouring sites
*wR*(*F*^2^) = 0.142	H-atom parameters constrained
*S* = 1.03	*w* = 1/[σ^2^(*F*_o_^2^) + (0.0615*P*)^2^ + 0.3342*P*] where *P* = (*F*_o_^2^ + 2*F*_c_^2^)/3
6250 reflections	(Δ/σ)_max_ < 0.001
244 parameters	Δρ_max_ = 0.23 e Å^−^^3^
0 restraints	Δρ_min_ = −0.19 e Å^−^^3^

### Special details

**Table:**

Geometry. All e.s.d.'s (except the e.s.d. in the dihedral angle between two l.s. planes) are estimated using the full covariance matrix. The cell e.s.d.'s are taken into account individually in the estimation of e.s.d.'s in distances, angles and torsion angles; correlations between e.s.d.'s in cell parameters are only used when they are defined by crystal symmetry. An approximate (isotropic) treatment of cell e.s.d.'s is used for estimating e.s.d.'s involving l.s. planes.
Refinement. Refinement of *F*^2^ against ALL reflections. The weighted *R*-factor *wR* and goodness of fit *S* are based on *F*^2^, conventional *R*-factors *R* are based on *F*, with *F* set to zero for negative *F*^2^. The threshold expression of *F*^2^ > σ(*F*^2^) is used only for calculating *R*-factors(gt) *etc*. and is not relevant to the choice of reflections for refinement. *R*-factors based on *F*^2^ are statistically about twice as large as those based on *F*, and *R*- factors based on ALL data will be even larger.

### Fractional atomic coordinates and isotropic or equivalent isotropic displacement parameters (Å^2^)

**Table:**

	*x*	*y*	*z*	*U*_iso_*/*U*_eq_	
C2	0.17408 (11)	0.62041 (6)	0.40308 (14)	0.0338 (3)	
H2	0.1120	0.6538	0.4087	0.041*	
C3	0.10682 (11)	0.57637 (7)	0.25999 (14)	0.0381 (3)	
C4	0.19953 (12)	0.53109 (7)	0.22723 (13)	0.0363 (3)	
C5	0.33023 (12)	0.55897 (7)	0.27795 (15)	0.0366 (3)	
H5A	0.3623	0.5459	0.2036	0.044*	
H5B	0.3812	0.5353	0.3709	0.044*	
C6	0.34585 (11)	0.63892 (6)	0.30324 (13)	0.0323 (2)	
H6	0.3138	0.6621	0.2048	0.039*	
C7	0.28230 (12)	0.73249 (7)	0.42790 (15)	0.0398 (3)	
H7	0.3400	0.7599	0.4081	0.048*	
C8	0.22360 (11)	0.58092 (7)	0.55153 (14)	0.0352 (3)	
C9	0.26574 (13)	0.51168 (7)	0.57097 (15)	0.0429 (3)	
H9	0.2638	0.4863	0.4880	0.052*	
C10	0.31075 (14)	0.47894 (8)	0.70986 (16)	0.0446 (3)	
H10	0.3396	0.4325	0.7196	0.054*	
C11	0.31275 (14)	0.51526 (8)	0.83330 (15)	0.0446 (3)	
C12	0.26964 (16)	0.58424 (8)	0.81650 (17)	0.0530 (4)	
H12	0.2700	0.6092	0.8993	0.064*	
C13	0.22628 (14)	0.61613 (8)	0.67819 (16)	0.0462 (3)	
H13	0.1979	0.6627	0.6690	0.055*	
C14	0.00369 (14)	0.53174 (9)	0.2744 (2)	0.0553 (4)	
H14A	−0.0532	0.5621	0.2953	0.083*	
H14B	0.0393	0.4983	0.3548	0.083*	
H14C	−0.0400	0.5067	0.1822	0.083*	
C15	0.04780 (14)	0.62642 (8)	0.12465 (17)	0.0513 (4)	
H15A	−0.0123	0.6563	0.1401	0.077*	
H15B	0.0076	0.5989	0.0351	0.077*	
H15C	0.1114	0.6553	0.1143	0.077*	
C16	0.48138 (11)	0.65875 (6)	0.38343 (14)	0.0332 (3)	
C17	0.55115 (11)	0.63584 (7)	0.52784 (14)	0.0368 (3)	
H17	0.5134	0.6082	0.5770	0.044*	
C18	0.67579 (12)	0.65292 (7)	0.60132 (16)	0.0418 (3)	
H18	0.7212	0.6366	0.6982	0.050*	
C19	0.73163 (13)	0.69426 (8)	0.52933 (19)	0.0511 (4)	
C20	0.66322 (15)	0.71813 (10)	0.3859 (2)	0.0623 (5)	
H20	0.7008	0.7464	0.3375	0.075*	
C21	0.53942 (14)	0.70045 (8)	0.31376 (17)	0.0488 (4)	
H21	0.4943	0.7168	0.2168	0.059*	
C22	0.3775 (2)	0.41457 (10)	0.9928 (2)	0.0736 (5)	
H22A	0.4042	0.4023	1.0969	0.110*	
H22B	0.4416	0.4027	0.9590	0.110*	
H22C	0.3032	0.3887	0.9349	0.110*	
C23	0.92890 (16)	0.68961 (11)	0.7357 (3)	0.0787 (6)	
H23A	1.0117	0.7079	0.7651	0.118*	
H23B	0.9311	0.6387	0.7350	0.118*	
H23C	0.8951	0.7053	0.8056	0.118*	
N1	0.27207 (9)	0.66443 (5)	0.38626 (11)	0.0321 (2)	
O1	0.17227 (10)	0.47451 (5)	0.16419 (12)	0.0515 (3)	
O2	0.22263 (10)	0.76191 (5)	0.48956 (13)	0.0524 (3)	
O3	0.35310 (13)	0.48797 (6)	0.97457 (12)	0.0646 (3)	
O4	0.85343 (11)	0.71456 (8)	0.59033 (17)	0.0816 (4)	

### Atomic displacement parameters (Å^2^)

**Table:**

	*U*^11^	*U*^22^	*U*^33^	*U*^12^	*U*^13^	*U*^23^
C2	0.0292 (5)	0.0337 (6)	0.0364 (6)	−0.0019 (5)	0.0109 (5)	−0.0052 (5)
C3	0.0320 (6)	0.0379 (6)	0.0374 (7)	−0.0076 (5)	0.0065 (5)	−0.0049 (5)
C4	0.0435 (7)	0.0340 (6)	0.0260 (6)	−0.0071 (5)	0.0080 (5)	−0.0030 (5)
C5	0.0382 (6)	0.0353 (6)	0.0354 (6)	−0.0023 (5)	0.0137 (5)	−0.0077 (5)
C6	0.0317 (6)	0.0327 (6)	0.0288 (6)	−0.0019 (5)	0.0081 (5)	−0.0006 (4)
C7	0.0364 (6)	0.0301 (6)	0.0448 (7)	−0.0007 (5)	0.0076 (6)	−0.0040 (5)
C8	0.0340 (6)	0.0369 (6)	0.0361 (6)	−0.0041 (5)	0.0155 (5)	−0.0055 (5)
C9	0.0548 (8)	0.0413 (7)	0.0374 (7)	0.0031 (6)	0.0234 (6)	−0.0050 (5)
C10	0.0556 (8)	0.0411 (7)	0.0425 (7)	0.0052 (6)	0.0252 (7)	0.0025 (6)
C11	0.0527 (8)	0.0492 (8)	0.0360 (7)	−0.0079 (6)	0.0220 (6)	−0.0009 (6)
C12	0.0787 (11)	0.0465 (8)	0.0418 (8)	−0.0045 (7)	0.0323 (8)	−0.0108 (6)
C13	0.0609 (9)	0.0382 (7)	0.0454 (8)	−0.0004 (6)	0.0275 (7)	−0.0072 (6)
C14	0.0413 (8)	0.0585 (9)	0.0620 (10)	−0.0193 (7)	0.0162 (7)	−0.0103 (8)
C15	0.0406 (7)	0.0506 (8)	0.0431 (8)	−0.0029 (6)	−0.0039 (6)	−0.0003 (6)
C16	0.0305 (5)	0.0305 (6)	0.0351 (6)	−0.0017 (4)	0.0094 (5)	0.0001 (5)
C17	0.0337 (6)	0.0379 (6)	0.0359 (6)	−0.0024 (5)	0.0108 (5)	0.0017 (5)
C18	0.0347 (6)	0.0400 (7)	0.0406 (7)	0.0003 (5)	0.0042 (5)	−0.0002 (5)
C19	0.0328 (7)	0.0457 (8)	0.0640 (10)	−0.0071 (6)	0.0081 (7)	0.0027 (7)
C20	0.0453 (8)	0.0647 (10)	0.0719 (11)	−0.0151 (7)	0.0182 (8)	0.0234 (9)
C21	0.0437 (8)	0.0492 (8)	0.0470 (8)	−0.0054 (6)	0.0113 (6)	0.0152 (6)
C22	0.1037 (15)	0.0640 (11)	0.0502 (10)	0.0027 (10)	0.0277 (10)	0.0144 (8)
C23	0.0342 (8)	0.0782 (13)	0.0957 (15)	−0.0048 (8)	−0.0036 (9)	0.0030 (11)
N1	0.0293 (5)	0.0279 (5)	0.0341 (5)	−0.0019 (4)	0.0076 (4)	−0.0031 (4)
O1	0.0621 (6)	0.0396 (5)	0.0481 (6)	−0.0136 (5)	0.0169 (5)	−0.0155 (4)
O2	0.0531 (6)	0.0367 (5)	0.0643 (7)	0.0024 (4)	0.0204 (5)	−0.0136 (5)
O3	0.1002 (9)	0.0585 (7)	0.0396 (6)	0.0004 (6)	0.0326 (6)	0.0052 (5)
O4	0.0371 (6)	0.0867 (9)	0.0976 (10)	−0.0224 (6)	0.0023 (6)	0.0220 (8)

### Geometric parameters (Å, °)

**Table:**

C2—N1	1.4785 (15)	C12—H12	0.9300
C2—C8	1.5181 (18)	C13—H13	0.9300
C2—C3	1.5395 (17)	C14—H14A	0.9600
C2—H2	0.9800	C14—H14B	0.9600
C3—C4	1.5128 (19)	C14—H14C	0.9600
C3—C14	1.5250 (19)	C15—H15A	0.9600
C3—C15	1.541 (2)	C15—H15B	0.9600
C4—O1	1.2081 (15)	C15—H15C	0.9600
C4—C5	1.5053 (18)	C16—C21	1.3811 (18)
C5—C6	1.5267 (17)	C16—C17	1.3818 (17)
C5—H5A	0.9700	C17—C18	1.3847 (18)
C5—H5B	0.9700	C17—H17	0.9300
C6—N1	1.4791 (15)	C18—C19	1.375 (2)
C6—C16	1.5117 (16)	C18—H18	0.9300
C6—H6	0.9800	C19—O4	1.3648 (17)
C7—O2	1.2211 (17)	C19—C20	1.377 (2)
C7—N1	1.3365 (16)	C20—C21	1.378 (2)
C7—H7	0.9300	C20—H20	0.9300
C8—C9	1.3821 (18)	C21—H21	0.9300
C8—C13	1.3877 (18)	C22—O3	1.410 (2)
C9—C10	1.3831 (19)	C22—H22A	0.9600
C9—H9	0.9300	C22—H22B	0.9600
C10—C11	1.3734 (19)	C22—H22C	0.9600
C10—H10	0.9300	C23—O4	1.415 (2)
C11—O3	1.3621 (17)	C23—H23A	0.9600
C11—C12	1.381 (2)	C23—H23B	0.9600
C12—C13	1.372 (2)	C23—H23C	0.9600
			
N1—C2—C8	111.15 (9)	C3—C14—H14A	109.5
N1—C2—C3	110.11 (10)	C3—C14—H14B	109.5
C8—C2—C3	117.24 (10)	H14A—C14—H14B	109.5
N1—C2—H2	105.8	C3—C14—H14C	109.5
C8—C2—H2	105.8	H14A—C14—H14C	109.5
C3—C2—H2	105.8	H14B—C14—H14C	109.5
C4—C3—C14	111.76 (11)	C3—C15—H15A	109.5
C4—C3—C2	110.01 (10)	C3—C15—H15B	109.5
C14—C3—C2	110.73 (11)	H15A—C15—H15B	109.5
C4—C3—C15	106.37 (11)	C3—C15—H15C	109.5
C14—C3—C15	108.28 (12)	H15A—C15—H15C	109.5
C2—C3—C15	109.55 (11)	H15B—C15—H15C	109.5
O1—C4—C5	120.22 (12)	C21—C16—C17	117.89 (12)
O1—C4—C3	122.55 (12)	C21—C16—C6	120.70 (11)
C5—C4—C3	117.22 (10)	C17—C16—C6	121.41 (11)
C4—C5—C6	116.02 (11)	C16—C17—C18	121.78 (12)
C4—C5—H5A	108.3	C16—C17—H17	119.1
C6—C5—H5A	108.3	C18—C17—H17	119.1
C4—C5—H5B	108.3	C19—C18—C17	119.24 (13)
C6—C5—H5B	108.3	C19—C18—H18	120.4
H5A—C5—H5B	107.4	C17—C18—H18	120.4
N1—C6—C16	111.21 (9)	O4—C19—C18	124.49 (15)
N1—C6—C5	110.49 (10)	O4—C19—C20	115.74 (14)
C16—C6—C5	111.18 (10)	C18—C19—C20	119.77 (13)
N1—C6—H6	107.9	C19—C20—C21	120.42 (14)
C16—C6—H6	107.9	C19—C20—H20	119.8
C5—C6—H6	107.9	C21—C20—H20	119.8
O2—C7—N1	125.80 (13)	C20—C21—C16	120.90 (14)
O2—C7—H7	117.1	C20—C21—H21	119.6
N1—C7—H7	117.1	C16—C21—H21	119.6
C9—C8—C13	116.76 (12)	O3—C22—H22A	109.5
C9—C8—C2	125.00 (11)	O3—C22—H22B	109.5
C13—C8—C2	118.23 (12)	H22A—C22—H22B	109.5
C8—C9—C10	122.10 (12)	O3—C22—H22C	109.5
C8—C9—H9	118.9	H22A—C22—H22C	109.5
C10—C9—H9	118.9	H22B—C22—H22C	109.5
C11—C10—C9	119.78 (13)	O4—C23—H23A	109.5
C11—C10—H10	120.1	O4—C23—H23B	109.5
C9—C10—H10	120.1	H23A—C23—H23B	109.5
O3—C11—C10	124.75 (14)	O4—C23—H23C	109.5
O3—C11—C12	116.01 (12)	H23A—C23—H23C	109.5
C10—C11—C12	119.23 (13)	H23B—C23—H23C	109.5
C13—C12—C11	120.24 (13)	C7—N1—C2	119.04 (10)
C13—C12—H12	119.9	C7—N1—C6	118.42 (10)
C11—C12—H12	119.9	C2—N1—C6	121.82 (9)
C12—C13—C8	121.86 (13)	C11—O3—C22	117.96 (12)
C12—C13—H13	119.1	C19—O4—C23	118.02 (14)
C8—C13—H13	119.1		
			
N1—C2—C3—C4	57.08 (13)	C2—C8—C13—C12	−179.98 (13)
C8—C2—C3—C4	−71.28 (13)	N1—C6—C16—C21	−120.55 (13)
N1—C2—C3—C14	−178.88 (11)	C5—C6—C16—C21	115.87 (14)
C8—C2—C3—C14	52.76 (15)	N1—C6—C16—C17	59.60 (15)
N1—C2—C3—C15	−59.52 (13)	C5—C6—C16—C17	−63.98 (15)
C8—C2—C3—C15	172.12 (11)	C21—C16—C17—C18	−0.8 (2)
C14—C3—C4—O1	26.68 (18)	C6—C16—C17—C18	179.06 (12)
C2—C3—C4—O1	150.13 (12)	C16—C17—C18—C19	0.5 (2)
C15—C3—C4—O1	−91.31 (15)	C17—C18—C19—O4	179.89 (15)
C14—C3—C4—C5	−152.76 (12)	C17—C18—C19—C20	0.1 (2)
C2—C3—C4—C5	−29.32 (15)	O4—C19—C20—C21	179.76 (17)
C15—C3—C4—C5	89.25 (13)	C18—C19—C20—C21	−0.4 (3)
O1—C4—C5—C6	158.68 (12)	C19—C20—C21—C16	0.2 (3)
C3—C4—C5—C6	−21.86 (16)	C17—C16—C21—C20	0.4 (2)
C4—C5—C6—N1	44.33 (14)	C6—C16—C21—C20	−179.41 (15)
C4—C5—C6—C16	168.32 (10)	O2—C7—N1—C2	5.3 (2)
N1—C2—C8—C9	−96.55 (14)	O2—C7—N1—C6	175.84 (12)
C3—C2—C8—C9	31.31 (17)	C8—C2—N1—C7	−93.80 (13)
N1—C2—C8—C13	84.08 (14)	C3—C2—N1—C7	134.58 (12)
C3—C2—C8—C13	−148.07 (12)	C8—C2—N1—C6	96.04 (12)
C13—C8—C9—C10	−1.2 (2)	C3—C2—N1—C6	−35.59 (14)
C2—C8—C9—C10	179.46 (13)	C16—C6—N1—C7	51.11 (14)
C8—C9—C10—C11	0.9 (2)	C5—C6—N1—C7	175.08 (11)
C9—C10—C11—O3	178.92 (14)	C16—C6—N1—C2	−138.67 (11)
C9—C10—C11—C12	−0.1 (2)	C5—C6—N1—C2	−14.70 (15)
O3—C11—C12—C13	−179.56 (15)	C10—C11—O3—C22	−9.8 (2)
C10—C11—C12—C13	−0.5 (2)	C12—C11—O3—C22	169.20 (16)
C11—C12—C13—C8	0.2 (2)	C18—C19—O4—C23	2.2 (3)
C9—C8—C13—C12	0.6 (2)	C20—C19—O4—C23	−178.04 (18)

### Hydrogen-bond geometry (Å, °)

**Table:**

*D*—H···*A*	*D*—H	H···*A*	*D*···*A*	*D*—H···*A*
C5—H5A···O3^i^	0.97	2.44	3.3446 (16)	155
C6—H6···O2^ii^	0.98	2.41	3.3708 (16)	168
C18—H18···O1^iii^	0.93	2.53	3.3018 (17)	140
C10—H10···Cg1^iv^	0.93	2.90	3.6627 (17)	140

Symmetry codes: (i) *x*, *y*, *z*−1; (ii) *x*, −*y*+3/2, *z*−1/2; (iii) −*x*+1, −*y*+1, −*z*+1; (iv) −*x*+1, −*y*, −*z*+2.
